# An empirical study on hospital-based prevention of recurrent urinary stone disease in Germany

**DOI:** 10.1007/s00345-021-03813-3

**Published:** 2021-08-18

**Authors:** Alina Reicherz, Patricia Rausch, Roman Herout, Joachim Noldus, Peter Bach

**Affiliations:** 1 Department of Urology, Marien Hospital Herne, Ruhr-University of Bochum, Hölkeskampring 40, 44625 Herne, Germany; 2 Department of Urology, University Carl Gustav Carus, Dresden, Germany

**Keywords:** Prevention, Recurrent urinary stone disease, Survey, Empirical study, Germany

## Abstract

**Purpose:**

Urinary stone disease is a common disease with a prevalence of 4.7% in Germany. The incidence increased over the last decades, and recurrence rates are up to 50% in the first 5 years after diagnosis. Adequate preventive measures can avoid up to 46% of stone recurrences. These numbers outline the importance of prevention. Especially among high-risk stone formers, specific diagnostics and measures are required. Published data indicate the divergence between the importance of prevention and its implementation in everyday clinical practice. This is the first survey among German urological departments highlighting medical care concerning the prevention of recurrent urinary stone disease, identifying challenges and providing recommendations for improvements.

**Methods:**

Two hundred and seventy urological hospital departments in Germany were anonymously surveyed about measurements to prevent recurrent stone disease. The questionnaire comprised 23 items dealing with diagnostics, counselling, knowledge among doctors concerning preventive measures and difficulties in preventing recurrent urinary stone disease.

**Results:**

Sixty-three urological departments (23.8%) answered the survey. The majority perform stone analysis at first and repeat events. Most patients with urinary stone disease receive general advice on preventive measures during their hospitalization. General recommendations focus on fluid intake and lifestyle changes. However, specific diets are infrequently recommended by inpatient urologists. Diagnostics to identify high-risk stone formers are mostly insufficient, and guideline-compliant urine tests are uncommon.

**Conclusion:**

The quality of secondary prevention needs to improve considerably. The focus should be put on identifying high-risk stone formers and offering those patients specific counselling. Furthermore, general advice on dietary recommendations should be extended.

**Supplementary Information:**

The online version contains supplementary material available at 10.1007/s00345-021-03813-3.

## Introduction

Urolithiasis is a common disease, 12% of the world's population suffers from urolithiasis once in a lifetime [1]. Over the last few decades, an increase in the incidence and prevalence rates was observed [2]. A continuous upward trend is expected due to a change in nutrition, lifestyle and rising temperatures caused by climate change [3]. The recurrence rate in stone patients is up to 50% within the first 5 years [4, 5]. Depending on the stone composition and causal disease, the recurrence rate can increase to 100% if no preventive measures are taken. The recurrence rate has increased twofold between 1979 and 2000 in Germany [5]. The observed and expected increase in patients with urinary calculi outlines the exigency to take action.

Nolde et al. showed that preventive measures can reduce relapse in up to 46% of stone patients [6]. Most stone patients require basic preventive measures comprising recommendations on fluid intake, diet and lifestyle changes. The German and European guidelines define groups at low and high risk for stone recurrence. About one quarter of patients has a high-risk profile. For high-risk stone formers, however, specific measures need to be taken to prevent a recurrence.

Recommendations are mainly based on experts’ opinions, as prospective randomized trials are rare [7]. In Germany, medical care concerning secondary prevention of urinary stones is mainly provided by specialized counselling hours in hospitals and outpatient clinics. However, it is not known to what extent it is implemented in daily routine. Prevention of urinary stone disease is poorly documented in the literature. A Canadian retrospective study on recurrent nephrolithiasis, published by Krepinsky et al. (2000), showed that in only 35.1% of patients presented for a metabolic investigation, a complete, guideline-compliant evaluation was performed [8]. A model calculation from 2006 showed that adequate diagnostics and prevention could save the German health care system about 178 million Euros per year [9].

This German-wide survey aims to analyze the current state of medical care regarding the prevention of stones, outline major challenges and provide recommendations to improve medical care.

## Methods

This survey is a German-wide empirical analysis evaluating the state of medical care concerning recurrent stone disease prevention. We designed a non-validated, anonymous questionnaire and sent it by email to directors of all German urology departments (*n* = 270) in February and March 2021. In the email, we asked for one physician to complete the questionnaire representatively. The questionnaire was designed using REDCap^®^. It comprised 23 questions dealing with metabolic evaluation, counselling of patients, and physicians' knowledge on preventive measures. We used Likert scale questions and multiple-choice questions. The answers matched recommendations of the German guidelines on urolithiasis [7].

Finally, we calculated descriptive statistics, including mean, standard deviation of the mean (SD), and interquartile range (IQR) for variables using Excel^®^, Version 2103.

## Results

In total, 63 of 270 urological departments responded to the survey and were included in the analysis. Five emails could not be delivered, resulting in an overall response rate of 23.8%.

Urological departments answering the questionnaire treat a total of 35,445 patients with urinary stone disease per year (cases estimated by responders). The mean number of patients treated with urinary stones per hospital is 572 (SD 358; IQR 300–700; estimation by responders). 28.6% of the responders were from academic hospitals.

### Stone analysis

The majority of the urological departments (50.8%) always analyze the stone’s composition in first-time stone formers; 22.2% responded that they mostly analyze it, 7.9% occasionally analyze it, 15.9% rarely analyze it, and 3.2% never analyze it. Repeat stone analysis is always performed in 49.2%. 11.5% answered they repeat stone analysis if recurrence occurs under pharmacological treatment, 21.3% repeat it if recurrence is shortly after interventional therapy with complete stone removal and 27.9% if relapse occurs after a prolonged stone-free period, while 8.2% never perform repeated stone analysis.

### Diagnostics

In patients with a high risk of recurrence, the majority of treating urologists (38.1%) rarely request repeated urinary pH measurements, while 9.5% never do so, 14.3% occasionally do, 17.5% mostly do, and 20.6% always include them in their diagnostic pathway.

For patients at high risk, 24-h urine collection is less often included in the standard diagnostic repertoire than repeat urinary pH measurements: 22.2% never, 30.2% rarely, 17.5% occasionally, 19.0% mostly, and 11.1% always perform a 24-h urine collection in patients with a high risk for recurrence.

Urologic departments were asked which blood test they run for patients with stones containing calcium oxalate or calcium phosphate. Results showed that blood laboratory analysis includes serum electrolytes in 93.5%, creatinine in 93.5%, uric acid in 77.4%, urea nitrogen in 82.3%, glomerular filtration rate in 80.6% and parathyroid hormone in 56.5%. 4.8% do not evaluate any of the parameters.

Hounsfield units indicating the stone's density can be included in the decision-making process for stone treatment. The handling differs between urologic departments: 14.3% never consider Hounsfield units, while 20.6% rarely do, 25.4% occasionally do, 22.2% mostly do, and 17.5% always do.

### Counselling

Urologic departments were asked if counselling for preventing recurrent urinary stone disease was mainly the outpatient clinic’s responsibility. 11.5% agreed that it is the duty of urologists in outpatient clinics; the majority (44.3%) mostly agreed, while 24.6% partially agreed and 19.7% disagreed.

In 54.1% of urological departments, urologists mostly and in 34.4% always give general advice during rounding or discussion upon discharge. Most departments (65.6%) do not offer an additional, specialized consultation hour for stone formers, while 19.7% offer an additional consultation hour for selected patients. In 14.8%, additional consultation hours are a regular part of medical care. Those clinics counsel on average about 57 patients per year [IQR: 20–50].

Urologists were asked to specify the content of counselling. General advice on intake of fluids comprises the amount (98.4%), circadian drinking (73.8%) and type of beverage (77.0%). Regarding nutrition, urologists mention a balanced diet (93.4%), protein intake (47.5%), salt intake (44.3%), oxalate intake (62.3%) and calcium intake (57.4%). Almost always (95.1%) lifestyle including activity, normalizing body weight and reducing stress are discussed.

Within counselling hours, the advice on fluid intake and nutrition is more comprehensive: 100% give recommendations on the amount, 81.0% on circadian drinking and 95.2% on the beverage type. Concerning nutrition, urologists discuss a balanced diet (90.5%), protein intake (81.0%), salt intake (76.2%), oxalate intake (85.7%) and calcium intake (85.7%). Commonly (95.2%), urologists advise on lifestyle.

Professional nutritional counselling is not standard of care considering that 4.9% answered they mostly offer, 27.9% occasionally offer, 37.7% rarely offer, and 29.5% never offer nutritional advice.

To assess the quality of preventive measures in German urological departments, we asked urologists if they agreed with the statement “counselling for patients with recurrent stone disease is excellent in our department”. Answers point out that urologists question their work: 6.6% agreed with the statement, the majority (47.5%) agreed partially, 27.9% agreed predominantly, and 18.0% disagreed.

### Risk factor

**Fig. 1:**
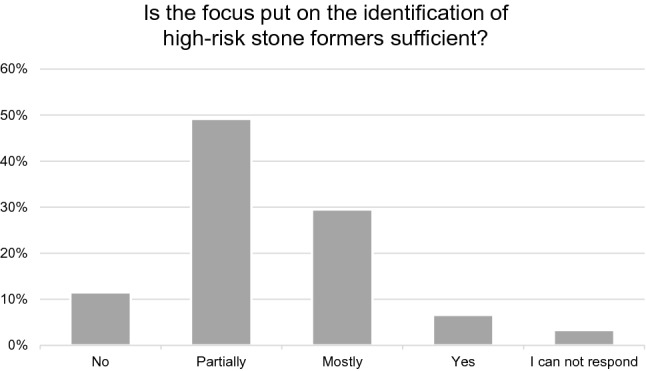
Identification of high-risk stone formers. *n* = 61, Likert scale (only one response possible)

Urologists were asked if they think that the attention drawn to risk factors like stone composition, family history, metabolic diseases and anatomic abnormalities is sufficient. The majority (49.2%) thought that the attention on these risk factors is partially sufficient, and 29.5% stated that it is predominantly sufficient (Fig. 1).

We tried to identify risk factors that are of little concern despite their importance. 77% agreed that more attention should be put on early occurrence (children or teenagers). Also, more attention should be turned on positive family history, stones containing urine acid, infectious stones and solitary kidneys (Table 1).

**Table 1:** Risk factors and associated diseases that are of little concern despite their importance, according to hospital-based urologists

Multiple choice question	
**Risk factors that are of little concern despite their importance**	
Early occurrence (in children or teenagers)	77.0% (47/61)
Positive family history	63.9% (39/61)
Brushite containing stones	31.1% (19/61)
Urine acid stones	54.1% (33/61)
Infectious stones	50.8% (31/61)
Single kidneys	47.5% (29/61)
None	3.3% (2/61)
**Associated diseases that are not given enough consideration**	
Hyperparathyroidism	70.5% (43/61)
Metabolic syndrome	63.9% (39/61)
Nephrocalcinosis	32.8% (20/61)
Polycystic kidney disease	16.4% (20/61)
Chronic gastroenteritis and bariatric surgery	67.2% (41/61)
Sarcoidosis	18.0% (11/61)
Neurogenic bladder dysfunction caused by spinal cord injuries	29.5% (18/61)
Cystinuria	36.1% (22/61)
Primary hyperoxaluria	44.3% (27/61)
Renal tubular acidosis	54.1% (33/61)
2,8-dihydroxyadeninuria	19.7% (12/61)
Xanthinuria	31.1% (19/61)
Lesch–Nyhan syndrome	16.4% (10/61)
Cystic fibrosis	21.3% (13/61)
None	3.3% (2/61)

We aimed to identify associated diseases that are not given enough consideration (Table 1). 70.5% of urologists answered that more attention should be paid to hyperparathyroidism in future. Responders also see an increased need for awareness about metabolic syndrome, nephrocalcinosis, chronic gastroenteritis and bariatric surgery, primary hyperoxaluria and renal tubular acidosis.

### Success rates of secondary prevention

We asked responders to estimate how many recurrent stone episodes can be prevented by good counselling on preventive measures. On average, urologists answered 36.2% (IQR 20–50) with a widespread range of 10–90%.

### Knowledge

The survey included two questions on the knowledge of preventive measures. Responders were asked to self-assess their knowledge and their co-workers’ knowledge in school grades (1–6; 1 corresponds to very good, and 6 to inadequate). On average, urologists rated themselves 2.6 (IQR 2–3) and their colleagues 2.9 (IQR 2–3).

### Additional comments

Responders remarked on the time intensity and insufficient reimbursement of diagnostics and stated that these factors cause neglect and result in inadequate training. Responders repeatedly commented on poor patients compliance concerning preventive measures.

## Discussion

Adequate diagnostic algorithms and preventive measures can reduce recurrent stone disease by 46% [6]. Per year 200,000 patients are estimated to have recurrent stone episodes in Germany [5]. Hypothetical that equals 80.000 patients experiencing avoidable stone recurrence. Nephrolithiasis is associated with an increased risk for chronic kidney disease and hypertension [10, 11]. Therefore, prevention is critical to prevent both an impact on quality of life and comorbidities. Moreover, it also has tremendous economic benefits; treatment costs could be lowered, and the loss of productive work time could be reduced [12]. Despite these facts, this survey outlined that diagnostics and patient counselling for urinary stone formers in Germany have room for improvement. To meet patients’ needs, urologists should tap the full potential of secondary prevention.

### Area of responsibility

To our knowledge, so far, no data existed on the extent of secondary prevention of urinary stones in German in- and outpatient clinics. Our survey showed that almost 90% (54/63) of urologic departments mostly or always give general advice during rounding or final discussion. Counselling for high-risk stone formers requires follow-up appointments for a specific metabolic workup. It is not reasonable during an acute stone event, as patients should be stone-free for at least 20 days before evaluation. Urologists working in hospitals regard counselling to prevent recurrent stone disease as the outpatient clinic's area of responsibility, and most departments do not offer an additional specialized consultation hour; academic hospitals offer it more often (33.3% vs. 6.7%). In our view, medical care would benefit from a discussion about who is to hold responsible for preventive measures. A first approach could be an adequate reimbursement. This is neither given in an outpatient nor in an inpatient clinic.

### General advice

Responders’ subjective grading of preventive measures in their urological department outlines that the quality is heterogeneous. While general advice on fluid intake and lifestyle, including activity, normalizing body weight and reducing stress is often sufficient, advice on a diet is incomplete. Protein, salt, oxalate and calcium intake are discussed in approximately half of the urologic departments during rounds. Patel et al. questioned members of the North Central Section of the American Urological Association and the Endourological Society on medical management of urolithiasis [13]. In comparison, they showed that 47% versus 69% recommend a low animal protein intake to all stone formers, while 3% versus 4% give recommendations only to calcium stone formers, 39% and 22% only to uric acid stone formers, and 10% versus 5% do not counsel their patients about low animal protein intake. One can speculate that different health care systems, especially legal and pecuniary differences, contribute to the difference. This is also evident from the “Additional Comments” of the survey mentioned in “Results“.

### High-risk stone formers

Stone analysis is the basis for further diagnostics, treatment plans and identification of high-risk stone formers [14]. The majority (46/63) of urological departments always or mostly analyze urinary stones at the first event. However, about 19% of the responders do not follow the guidelines’ recommendations. They stated that their department never or rarely analyzed urinary stones. To detect a change in the composition, the majority perform stone analysis in repeated stone events.

General advice concerning preventive measures is recommended by German, European and American guidelines, while an extended diagnostic algorithm based on the composition of the stone is required for stone formers at high risk to rule out associated metabolic diseases [7, 14, 15]. In 2014 an American retrospective study evaluated the prevalence of 24-h urine testing among patients at high risk for recurrence; it was 7.4% among 28,836 patients identified [16]. Our survey shows that in Germany, repeated urine pH measurements and 24-h urine collection are also not routinely requested.

Necessary blood tests for patients with stones containing calcium oxalate or calcium phosphate are mostly run according to the guidelines. However, about 45% do not evaluate parathyroid hormone, and more than 20% do not evaluate uric acid.

About 25% of the patients who form stones are at high risk for recurrence [17]. Counselling should be offered to those patients. This survey outlines the challenges we face; urological departments counsel only 3.9% (estimation by responders) of all patients they treat with urinary stone disease. This equals an estimated one in six high-risk patients who undergoes an extended diagnostic algorithm.

Results of the survey demonstrated urologists' lack of awareness for risk factors (Fig. 1). Only about one-quarter of the urologists thought that the attention drawn to risk factors like stone composition, family history, metabolic diseases and anatomic abnormalities are predominantly or entirely sufficient. Responders agreed that there is a wide range of risk factors and associated diseases of little concern despite their importance (Table 1). The included self-assessment results show that urologists are only “satisfied” with their knowledge and their colleagues' knowledge on preventive measures (in German school grades 3 + to 3, corresponds to c in the American grading). This finding is not surprising. In a survey by Wertheim et al. from 2013 on nutrition in the management of urinary stones, members of the Endourological Society were questioned about providing nutrition recommendations. 52% of endourologists reported having low confidence in providing specific strategies to achieve optimal calcium intake [18].

Our results show that professional nutritional counselling is not standard of care in Germany. However, to improve counselling, nutritionists could be further included in medical care. This would bridge gaps of knowledge and lack of time in daily hospital practice. A survey distributed to American registered dieticians revealed that few stone formers are provided with nutritional counselling. About 50% of dieticians provide medical, nutritional counselling for urolithiasis, but 80% of them estimated that they see only one to two patients per month [19].

Reasons for neglect of secondary prevention of urinary stone disease should be discussed. Among improved techniques of endourological stone removal, a lack of time and low financial incentives, a wrong understanding of the patients’ demands might be potential reasons. A survey among 159 stone formers and endourologists regarding the demand found that “most patients with stones will consider preventive medical therapy to avoid recurrent pain or a surgical procedure. In contrast, most urologists perceive that patients prefer to avoid medication even if it means tolerating several acute stone events and/or surgical procedures” [20].

Our study has the following limitations. First, the questionnaire is non-validated. This limits the reliability and validity of findings. A second limitation is that because the response rate is low it might be underpowered and pose a potential bias. However, in comparison, the survey on the medical management of urolithiasis among members of the North Central Section of the American Urological Association and the Endourological Society achieved a response rate of 14.4% and 9.1%, respectively [13].

Our study provides relevant data as there is no record available on the actions taken to prevent stones in Germany. A questionnaire that explicitly tackles this area of stone disease does not exist.

We draw three conclusions from the survey: first, urologists must take responsibility for not only treating but also preventing recurrent stone disease. To achieve a balanced responsibility between inpatient and outpatient clinics, adequate reimbursement is essential. Second, urologic departments should extend their general advice on dietary considerations. Additional nutritional counselling by dieticians would fill a gap in urologists' knowledge and lack of time. And third, we need to improve the identification of patients at high risk for recurrence and apply diagnostic algorithms in those patients.

## Supplementary Information

Below is the link to the electronic supplementary material.

Supplementary file1 (DOCX 30 KB)
