# Supplementary material for: An empirical study on hospital-based prevention of recurrent urinary stone disease in Germany
Source: World J Urol. 2021 Aug 18;40(1):237–42. doi: 10.1007/s00345-021-03813-3 (PMC8813807; doi:10.1007/s00345-021-03813-3)
Supplement: Supplementary file 1 — Supplementary file1 (DOCX 30 KB) [file 345_2021_3813_MOESM1_ESM.docx]

# Supplementary

|  | Mean | SD | IQR |
| --- | --- | --- | --- |
| Patients treated with urinary calculi per year (Estimation) | 571.7 | 358.3 | 300 – 700 |

|  |  | % | n = |
| --- | --- | --- | --- |
| Academic hospital | Yes  No | 28.6  71.4 | 18  45 |

|  | Never | Rarely | Occasionally | Mostly | Always |
| --- | --- | --- | --- | --- | --- |
| Stone analysis at first event | 3.2% (2) | 15.9% (10) | 7.9% (5) | 22.2% (14) | 50.8% (32) |

|  | No | Recurrence under pharmacological prevention | Early recurrence after interventional therapy with complete stone clearance | Late recurrence after a prolonged stone-free period | Always |
| --- | --- | --- | --- | --- | --- |
| Stone analysis at repeated event | 8.2% (5) | 11.5% (7) | 21.3% (13) | 27.9% (17) | 49.2% (30) |

|  | Never | Rarely | Occasionally | Mostly | Always |
| --- | --- | --- | --- | --- | --- |
| Urine-pH profile in high-risk stone formers | 9.5% (6) | 38.1% (24) | 14.3% (9) | 17.5% (11) | 20.6% (13) |
| 24-h urine collection in high risk stone formers | 22.2% (14) | 30.2% (19) | 17.5% (11) | 19.0% (12) | 11.1% (7) |

|  | None | Glomerulary filtration rate | Parathyroid hormone | Serum electrolytes | Uric acid | Urea nitrogen | Creatinine |
| --- | --- | --- | --- | --- | --- | --- | --- |
| Bloodwork in calcium oxalate/phosphate stone formers | 4.8% (3) | 80.6% (50) | 56.5% (35) | 93.5% (58) | 77.4% (48) | 82.3% (51) | 93.5% (58) |

|  | Never | Rarely | Occasionally | Mostly | Always |
| --- | --- | --- | --- | --- | --- |
| Is the stone density (Hounsfield units) considered for the planning of therapy? | 14.3% (9) | 20.6% (13) | 25.4% (16) | 22.2% (14) | 17.5% (11) |

|  | No | Partially | Mostly | Yes |
| --- | --- | --- | --- | --- |
| Responder agreed that counselling on preventive measures is the responsibility of outpatient clinics. | 19.7% (12) | 24.6% (15) | 44.3% (27) | 11.5% (7) |

|  | Never | Rarely | Occasionally | Mostly | Always |
| --- | --- | --- | --- | --- | --- |
| I explain general preventive measures during rounding/final discussion. | 0% (0) | 1.6% (1) | 9.8% (6) | 54.1% (33) | 34.4% (11) |

|  | No | In individual cases | Yes |
| --- | --- | --- | --- |
| We offer a specialized counselling for stone patients. | 65.6% (40) | 19.7% (12) | 14.8% (9) |

|  | Mean | SD | IQR |
| --- | --- | --- | --- |
| Estimated number of patients counselled in a specialized hour for stone formers. | 57.4 | 64.2 | 20 - 50 |

|  | General advice during rounding/upon discharge | General advice in specialized counselling |
| --- | --- | --- |
| Fluid uptake | | |
| - Amount | 98.4% (60) | 100% (21) |
| - Circadian drinking | 73.8% (45) | 81.0% (17) |
| - Type of beverage | 77.0% (47) | 95.2% (20) |
| Nutrition | | |
| - Balanced diet | 93.4% (57) | 90.5% (19) |
| - Protein intake | 47.5% (29) | 81.0% (17) |
| - Salt intake | 44.3% (27) | 76.2% (16) |
| - Oxalate intake | 62.3% (38) | 85.7% (18) |
| - Calcium intake | 57.4% (35) | 85.7% (18) |
| Lifestyle | 95.1% (58) | 95.2% (20) |

|  | Never | Rarely | Occasionally | Mostly | Always |
| --- | --- | --- | --- | --- | --- |
| Professional nutritional advice offered | 29.5% (18) | 37.7% (23) | 27.9% (17) | 4.9% (3) | 0% (0) |

|  | Risk factors that are of little concern despite their importance |
| --- | --- |
| Early occurrence (in children or teenagers) | 77% (47) |
| Positive family history | 63.9% (39) |
| Brushite containing stones | 31.1% (19) |
| Urine acid stones | 54.1% (33) |
| Infectious stones | 50.8% (31) |
| Single kidneys | 47.5% (29) |
| None | 3.3% (2) |

|  | Associated diseases that are not given enough consideration |
| --- | --- |
| Hyperparathyroidism | 70.5% (43) |
| Metabolic syndrome | 63.9% (39) |
| Nephrocalcinosis | 32.8% (20) |
| Polycystic kidney disease | 32.8% (20) |
| Chronic gastroenteritis and bariatric surgery | 67.2% (41) |
| Sarcoidosis | 18.0% (11) |
| Neurogenic bladder dysfunction caused by spinal cord injuries | 29.5% (18) |
| Cystinuria | 36.1% (22) |
| Primary hyperoxaluria | 44.3% (27) |
| Renal tubular acidosis | 54.1% (33) |
| 2,8-dihydroxyadeninuria | 19.7% (12) |
| Xanthinuria | 31.1% (19) |
| Lesch–Nyhan syndrome | 16.4% (10) |
| Cystic fibrosis | 21.3% (13) |
| None | 3.3% (2) |

|  | No | Partially | Mostly | Yes | I can not respond. |
| --- | --- | --- | --- | --- | --- |
| Is the focus put on the identification of high-risk stone formers sufficient? | 11.5% (7) | 49.2% (30) | 29.5% (18) | 6.6% (4) | 3.3% (2) |

|  | Mean | SD | IQR |
| --- | --- | --- | --- |
| Percentage of urinary stones that could be prevented by specialized counselling (estimated by the responder). | 36.2 | 21.2 | 20 – 50 |

|  | Mean | SD | IQR |
| --- | --- | --- | --- |
| Responders knowledge on preventive measures | 2.6 | 1.0 | 2 – 3 |
| Responder's co-workers' knowledge on preventive measures | 2.9 | 1.1 | 2 – 3 |

|  | No | Partially | Mostly | Yes |
| --- | --- | --- | --- | --- |
| Responder agreed that counselling on preventive measures is very good in her/his clinic. | 18.0% (11) | 47.5% (29) | 27.9% (17) | 6.6% (4) |
